# Supplementary material for: The Gut Microbiome Regulates the Psychomotor Effects and Context-Dependent Rewarding Responses to Cocaine in Germ-Free and Antibiotic-Treated Animal Models
Source: Microorganisms. 2025 Jan 3;13(1):77. doi: 10.3390/microorganisms13010077 (PMC11767876; doi:10.3390/microorganisms13010077)
Supplement: Supplementary file 1 [file microorganisms-13-00077-s001.zip › microorganisms-3407947-supplementary.pdf]

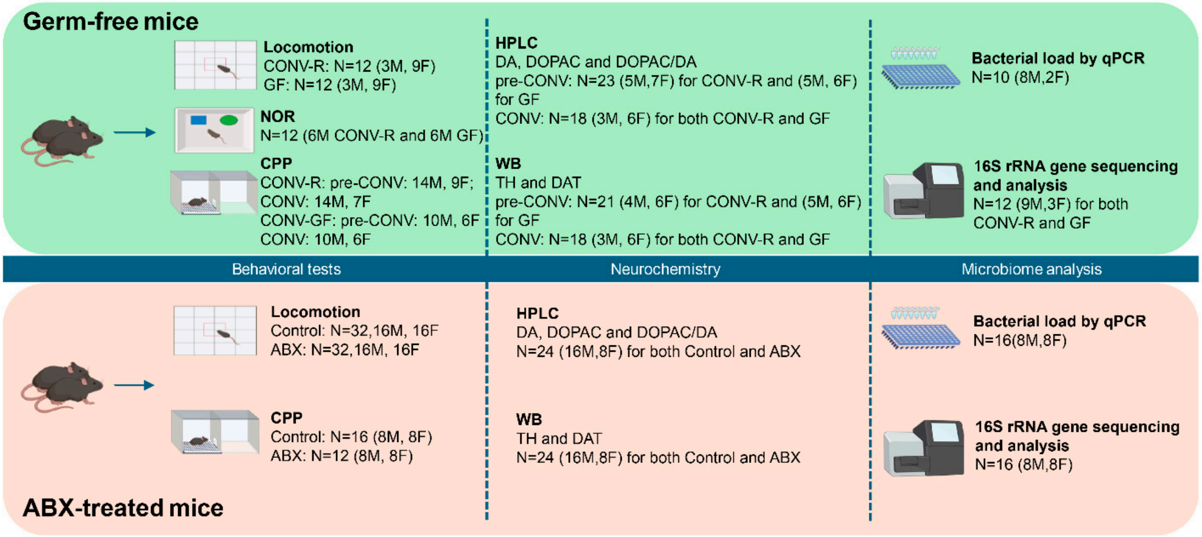

Figure S1. Experimental design and sample sizes for assessing behavioral, neurochemical, and microbiome outcomes in germ-free (GF) and antibiotic-treated (ABX) mice. Behavioral tests: locomotion, Novel Object Recognition (NOR), and conditioned place preference (CPP) were evaluated across experimental groups. Neurochemistry: high-performance liquid chromatography (HPLC) was used to measure dopamine (DA), 3,4-dihydroxyphenylacetic acid (DOPAC), and DOPAC/DA ratios. Western blot (WB) analyses targeted tyrosine hydroxylase (TH) and dopamine transporter (DAT). Microbiome analysis: bacterial load was quantified using qPCR, and 16S rRNA gene sequencing was performed for microbiome profiling.
